# *N*′-(4-Hy­droxy­benzyl­idene)-3-meth­oxy­benzohydrazide

**DOI:** 10.1107/S1600536811055565

**Published:** 2012-01-07

**Authors:** Chun-Bao Tang

**Affiliations:** a Department of Chemistry, Jiaying University, Meizhou 514015, People’s Republic of China

## Abstract

In the title compound, C_15_H_14_N_2_O_3_, the dihedral angle between the two benzene rings is 47.9 (3)°. In the crystal, mol­ecules are linked through N—H⋯O, O—H⋯O and O—H⋯N hydrogen bonds, forming layers parallel to the *ab* plane.

## Related literature

For general background to hydrazones, see: Rasras *et al.* (2010 ▶); Pyta *et al.* (2010 ▶); Angelusiu *et al.* (2010 ▶). For related structures, see: Fun *et al.* (2008 ▶); Singh & Singh (2010 ▶); Ahmad *et al.* (2010 ▶); Tang (2010 ▶, 2011 ▶). For reference bond-length data, see: Allen *et al.* (1987 ▶).
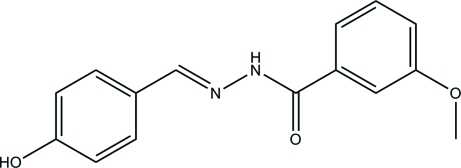


## Experimental

### 

#### Crystal data

C_15_H_14_N_2_O_3_
*M* *_r_* = 270.28
Orthorhombic, 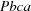

*a* = 13.221 (2) Å
*b* = 9.5336 (18) Å
*c* = 21.620 (2) Å
*V* = 2725.1 (7) Å^3^
*Z* = 8
Mo *K*α radiation
μ = 0.09 mm^−1^
*T* = 298 K
0.23 × 0.21 × 0.20 mm

#### Data collection

Bruker SMART CCD area-detector diffractometer
Absorption correction: multi-scan (*SADABS*; Sheldrick, 1996 ▶) *T*_min_ = 0.979, *T*_max_ = 0.982
18234 measured reflections
2531 independent reflections
1805 reflections with *I* > 2σ(*I*)
*R*_int_ = 0.069

#### Refinement

*R*[*F* ^2^ > 2σ(*F* ^2^)] = 0.061
*wR*(*F* ^2^) = 0.117
*S* = 1.11
2531 reflections
188 parameters
2 restraints
H atoms treated by a mixture of independent and constrained refinement
Δρ_max_ = 0.18 e Å^−3^
Δρ_min_ = −0.20 e Å^−3^

### 

Data collection: *SMART* (Bruker, 2002 ▶); cell refinement: *SAINT* (Bruker, 2002 ▶); data reduction: *SAINT*; program(s) used to solve structure: *SHELXS97* (Sheldrick, 2008 ▶); program(s) used to refine structure: *SHELXL97* (Sheldrick, 2008 ▶); molecular graphics: *SHELXTL* (Sheldrick, 2008 ▶); software used to prepare material for publication: *SHELXL97*.

## Supplementary Material

Crystal structure: contains datablock(s) global, I. DOI: 10.1107/S1600536811055565/cv5225sup1.cif

Structure factors: contains datablock(s) I. DOI: 10.1107/S1600536811055565/cv5225Isup2.hkl

Supplementary material file. DOI: 10.1107/S1600536811055565/cv5225Isup3.cml

Additional supplementary materials:  crystallographic information; 3D view; checkCIF report

## Figures and Tables

**Table 1:** Hydrogen-bond geometry (Å, °)

*D*—H⋯*A*	*D*—H	H⋯*A*	*D*⋯*A*	*D*—H⋯*A*
N2—H2⋯O2^i^	0.90 (1)	2.13 (1)	3.000 (2)	162 (3)
O1—H1⋯N1^ii^	0.85 (1)	2.11 (1)	2.959 (2)	176 (3)
O1—H1⋯O2^ii^	0.85 (1)	2.53 (3)	2.969 (2)	113 (3)

Symmetry codes: (i) 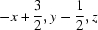; (ii) 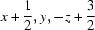.

**Fig. 1.**
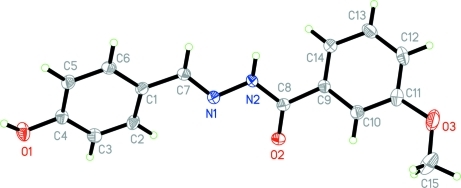
The molecular structure of the compound, showing the atom-numbering scheme. Displacement ellipsoids are drawn at the 30% probability level.

## supplementary crystallographic information

### Comment

Hydrazone compounds have received much attention in biological and structural chemistry in the last years (Rasras *et al.*, 2010; Pyta *et al.*, 2010; Angelusiu *et al.*, 2010; Fun *et al.*, 2008; Singh & Singh, 2010; Ahmad *et al.*, 2010). As a continuation of our work on the structural study on such compounds (Tang, 2010, 2011), the author reports herein the crystal structure of the title new hydrazone compound (Fig. 1).

In the molecule of the title compound, the dihedral angle between the two benzene rings is 47.9 (3)°. Bond lengths in the compound are normal (Allen *et al.*, 1987) and comparable to those in the similar compounds the author reported previously. In the crystal structure, molecules are linked through intermolecular N—H···O, O—H···O, and O—H···N hydrogen bonds (Table 1), forming layers parallel to the *ab* plane.

### Experimental

4-Hydroxybenzaldehyde (0.1 mmol, 12.2 mg) and 3-methoxybenzohydrazide (0.1 mmol, 16.6 mg) were dissolved in methanol (20 ml). The mixture was stirred at reflux for 10 min to give a clear colourless solution. Colourless needle-shaped crystals of the compound were formed by slow evaporation of the solvent over several days.

### Refinement

The amino and hydroxyl H atoms were located in a difference Fourier map and refined isotropically, with the O—H and N—H distances restrained to 0.85 (1) and 0.90 (1) Å, respectively. Other H atoms were constrained to ideal geometries and refined as riding, with Csp^2^—H = 0.93 Å, and C(methyl)—H = 0.96 Å; *U*_iso_(H) = 1.2*U*_eq_(C) and 1.5*U*_eq_(C_methyl_).

### Crystal data

**Table:**

C_15_H_14_N_2_O_3_	*D*_x_ = 1.318 Mg m^−^^3^
*M**_r_* = 270.28	Mo *K*α radiation, λ = 0.71073 Å
Orthorhombic, *P**b**c**a*	Cell parameters from 2706 reflections
*a* = 13.221 (2) Å	θ = 2.4–24.6°
*b* = 9.5336 (18) Å	µ = 0.09 mm^−^^1^
*c* = 21.620 (2) Å	*T* = 298 K
*V* = 2725.1 (7) Å^3^	Cut from needle, colorless
*Z* = 8	0.23 × 0.21 × 0.20 mm
*F*(000) = 1136	

### Data collection

**Table:**

Bruker SMART CCD area-detector diffractometer	2531 independent reflections
Radiation source: fine-focus sealed tube	1805 reflections with *I* > 2σ(*I*)
graphite	*R*_int_ = 0.069
ω scans	θ_max_ = 25.5°, θ_min_ = 2.4°
Absorption correction: multi-scan (*SADABS*; Sheldrick, 1996)	*h* = −16→16
*T*_min_ = 0.979, *T*_max_ = 0.982	*k* = −11→11
18234 measured reflections	*l* = −22→26

### Refinement

**Table:**

Refinement on *F*^2^	Primary atom site location: structure-invariant direct methods
Least-squares matrix: full	Secondary atom site location: difference Fourier map
*R*[*F*^2^ > 2σ(*F*^2^)] = 0.061	Hydrogen site location: inferred from neighbouring sites
*wR*(*F*^2^) = 0.117	H atoms treated by a mixture of independent and constrained refinement
*S* = 1.11	*w* = 1/[σ^2^(*F*_o_^2^) + (0.041*P*)^2^ + 0.712*P*] where *P* = (*F*_o_^2^ + 2*F*_c_^2^)/3
2531 reflections	(Δ/σ)_max_ < 0.001
188 parameters	Δρ_max_ = 0.18 e Å^−^^3^
2 restraints	Δρ_min_ = −0.20 e Å^−^^3^

### Special details

**Table:**

Geometry. All esds (except the esd in the dihedral angle between two l.s. planes) are estimated using the full covariance matrix. The cell esds are taken into account individually in the estimation of esds in distances, angles and torsion angles; correlations between esds in cell parameters are only used when they are defined by crystal symmetry. An approximate (isotropic) treatment of cell esds is used for estimating esds involving l.s. planes.
Refinement. Refinement of F^2^ against ALL reflections. The weighted R-factor wR and goodness of fit S are based on F^2^, conventional R-factors R are based on F, with F set to zero for negative F^2^. The threshold expression of F^2^ > 2sigma(F^2^) is used only for calculating R-factors(gt) etc. and is not relevant to the choice of reflections for refinement. R-factors based on F^2^ are statistically about twice as large as those based on F, and R- factors based on ALL data will be even larger.

### Fractional atomic coordinates and isotropic or equivalent isotropic displacement parameters (Å^2^)

**Table:**

	*x*	*y*	*z*	*U*_iso_*/*U*_eq_	
N1	0.84363 (13)	0.96516 (19)	0.63698 (8)	0.0335 (5)	
N2	0.76637 (13)	0.89157 (18)	0.60808 (9)	0.0331 (5)	
O1	1.24869 (13)	1.15604 (17)	0.77207 (8)	0.0491 (5)	
O2	0.67126 (11)	1.08925 (16)	0.60246 (8)	0.0416 (4)	
O3	0.31857 (12)	0.8832 (2)	0.55817 (10)	0.0676 (6)	
C1	1.00898 (16)	0.9639 (2)	0.68058 (11)	0.0327 (5)	
C2	1.02194 (17)	1.1091 (2)	0.67981 (12)	0.0390 (6)	
H2A	0.9757	1.1648	0.6586	0.047*	
C3	1.10217 (17)	1.1714 (2)	0.70994 (11)	0.0414 (6)	
H3	1.1102	1.2682	0.7084	0.050*	
C4	1.17085 (16)	1.0898 (2)	0.74251 (11)	0.0340 (6)	
C5	1.16016 (17)	0.9463 (2)	0.74303 (11)	0.0378 (6)	
H5	1.2064	0.8911	0.7645	0.045*	
C6	1.08096 (16)	0.8842 (2)	0.71180 (12)	0.0384 (6)	
H6	1.0755	0.7869	0.7116	0.046*	
C7	0.92309 (16)	0.8958 (2)	0.65072 (11)	0.0338 (6)	
H7	0.9263	0.8005	0.6416	0.041*	
C8	0.68050 (16)	0.9628 (2)	0.59326 (10)	0.0311 (5)	
C9	0.59785 (15)	0.8768 (2)	0.56562 (10)	0.0300 (5)	
C10	0.49867 (16)	0.9232 (2)	0.57510 (11)	0.0365 (6)	
H10	0.4868	1.0042	0.5980	0.044*	
C11	0.41857 (17)	0.8491 (3)	0.55060 (12)	0.0429 (6)	
C12	0.43747 (19)	0.7307 (3)	0.51536 (13)	0.0495 (7)	
H12	0.3837	0.6808	0.4985	0.059*	
C13	0.53502 (19)	0.6863 (3)	0.50505 (12)	0.0463 (7)	
H13	0.5467	0.6076	0.4807	0.056*	
C14	0.61629 (17)	0.7580 (2)	0.53071 (10)	0.0375 (6)	
H14	0.6821	0.7266	0.5245	0.045*	
C15	0.2946 (2)	1.0000 (4)	0.59606 (15)	0.0752 (10)	
H15A	0.3231	0.9867	0.6365	0.113*	
H15B	0.2224	1.0087	0.5994	0.113*	
H15C	0.3219	1.0838	0.5779	0.113*	
H2	0.770 (2)	0.7974 (11)	0.6051 (14)	0.090*	
H1	1.276 (2)	1.104 (3)	0.7996 (13)	0.113*	

### Atomic displacement parameters (Å^2^)

**Table:**

	*U*^11^	*U*^22^	*U*^33^	*U*^12^	*U*^13^	*U*^23^
N1	0.0297 (10)	0.0310 (10)	0.0398 (12)	−0.0013 (9)	−0.0022 (9)	−0.0029 (9)
N2	0.0288 (10)	0.0267 (10)	0.0439 (12)	−0.0026 (9)	−0.0047 (9)	−0.0049 (9)
O1	0.0510 (10)	0.0357 (10)	0.0607 (13)	−0.0157 (8)	−0.0214 (10)	0.0127 (9)
O2	0.0395 (9)	0.0267 (9)	0.0587 (12)	0.0026 (7)	−0.0059 (8)	−0.0060 (8)
O3	0.0298 (10)	0.0944 (16)	0.0784 (15)	−0.0011 (10)	−0.0039 (10)	−0.0058 (13)
C1	0.0298 (12)	0.0289 (12)	0.0393 (14)	0.0024 (10)	0.0001 (11)	0.0002 (11)
C2	0.0347 (13)	0.0336 (13)	0.0486 (16)	0.0032 (11)	−0.0085 (11)	0.0082 (12)
C3	0.0468 (15)	0.0241 (12)	0.0532 (17)	−0.0039 (11)	−0.0067 (13)	0.0065 (12)
C4	0.0332 (12)	0.0319 (12)	0.0370 (14)	−0.0054 (10)	−0.0027 (11)	0.0053 (11)
C5	0.0375 (13)	0.0275 (12)	0.0483 (16)	0.0020 (10)	−0.0085 (12)	0.0070 (11)
C6	0.0391 (13)	0.0218 (12)	0.0544 (17)	−0.0015 (10)	−0.0041 (12)	0.0001 (11)
C7	0.0346 (13)	0.0261 (12)	0.0406 (15)	−0.0007 (10)	−0.0007 (11)	−0.0012 (11)
C8	0.0320 (12)	0.0305 (13)	0.0307 (14)	0.0004 (10)	0.0033 (10)	0.0018 (11)
C9	0.0305 (12)	0.0274 (12)	0.0322 (14)	−0.0029 (9)	−0.0020 (10)	0.0049 (11)
C10	0.0357 (13)	0.0350 (13)	0.0389 (15)	0.0000 (10)	−0.0014 (11)	0.0012 (11)
C11	0.0317 (13)	0.0542 (16)	0.0427 (16)	−0.0045 (12)	−0.0032 (12)	0.0085 (13)
C12	0.0420 (15)	0.0547 (17)	0.0518 (18)	−0.0145 (13)	−0.0148 (13)	0.0015 (14)
C13	0.0521 (16)	0.0382 (14)	0.0486 (17)	−0.0050 (12)	−0.0090 (14)	−0.0092 (12)
C14	0.0356 (13)	0.0344 (13)	0.0426 (15)	−0.0008 (11)	−0.0021 (11)	−0.0028 (12)
C15	0.0442 (17)	0.107 (3)	0.074 (2)	0.0198 (17)	0.0123 (16)	0.008 (2)

### Geometric parameters (Å, °)

**Table:**

N1—C7	1.277 (3)	C5—H5	0.9300
N1—N2	1.388 (2)	C6—H6	0.9300
N2—C8	1.361 (3)	C7—H7	0.9300
N2—H2	0.901 (10)	C8—C9	1.491 (3)
O1—C4	1.366 (3)	C9—C14	1.382 (3)
O1—H1	0.853 (10)	C9—C10	1.399 (3)
O2—C8	1.228 (2)	C10—C11	1.379 (3)
O3—C11	1.371 (3)	C10—H10	0.9300
O3—C15	1.419 (3)	C11—C12	1.384 (4)
C1—C6	1.392 (3)	C12—C13	1.376 (3)
C1—C2	1.395 (3)	C12—H12	0.9300
C1—C7	1.459 (3)	C13—C14	1.389 (3)
C2—C3	1.379 (3)	C13—H13	0.9300
C2—H2A	0.9300	C14—H14	0.9300
C3—C4	1.387 (3)	C15—H15A	0.9600
C3—H3	0.9300	C15—H15B	0.9600
C4—C5	1.376 (3)	C15—H15C	0.9600
C5—C6	1.379 (3)		
			
C7—N1—N2	116.64 (18)	O2—C8—N2	122.3 (2)
C8—N2—N1	117.87 (17)	O2—C8—C9	122.15 (19)
C8—N2—H2	121.7 (19)	N2—C8—C9	115.51 (19)
N1—N2—H2	119.8 (19)	C14—C9—C10	120.3 (2)
C4—O1—H1	112 (2)	C14—C9—C8	122.69 (19)
C11—O3—C15	118.1 (2)	C10—C9—C8	117.0 (2)
C6—C1—C2	117.6 (2)	C11—C10—C9	120.1 (2)
C6—C1—C7	120.3 (2)	C11—C10—H10	120.0
C2—C1—C7	122.1 (2)	C9—C10—H10	120.0
C3—C2—C1	121.1 (2)	O3—C11—C10	125.0 (2)
C3—C2—H2A	119.5	O3—C11—C12	115.7 (2)
C1—C2—H2A	119.5	C10—C11—C12	119.4 (2)
C2—C3—C4	120.1 (2)	C13—C12—C11	120.6 (2)
C2—C3—H3	119.9	C13—C12—H12	119.7
C4—C3—H3	119.9	C11—C12—H12	119.7
O1—C4—C5	122.2 (2)	C12—C13—C14	120.6 (2)
O1—C4—C3	118.13 (19)	C12—C13—H13	119.7
C5—C4—C3	119.6 (2)	C14—C13—H13	119.7
C4—C5—C6	120.1 (2)	C9—C14—C13	119.0 (2)
C4—C5—H5	120.0	C9—C14—H14	120.5
C6—C5—H5	120.0	C13—C14—H14	120.5
C5—C6—C1	121.5 (2)	O3—C15—H15A	109.5
C5—C6—H6	119.3	O3—C15—H15B	109.5
C1—C6—H6	119.3	H15A—C15—H15B	109.5
N1—C7—C1	120.9 (2)	O3—C15—H15C	109.5
N1—C7—H7	119.6	H15A—C15—H15C	109.5
C1—C7—H7	119.6	H15B—C15—H15C	109.5

### Hydrogen-bond geometry (Å, °)

**Table:**

*D*—H···*A*	*D*—H	H···*A*	*D*···*A*	*D*—H···*A*
N2—H2···O2^i^	0.90 (1)	2.13 (1)	3.000 (2)	162 (3)
O1—H1···N1^ii^	0.85 (1)	2.11 (1)	2.959 (2)	176 (3)
O1—H1···O2^ii^	0.85 (1)	2.53 (3)	2.969 (2)	113 (3)

Symmetry codes: (i) −*x*+3/2, *y*−1/2, *z*; (ii) *x*+1/2, *y*, −*z*+3/2.
